# Supplementary material for: Chromatin topology reorganization and transcription repression by PML-RARα in acute promyeloid leukemia
Source: Genome Biol. 2020 May 11;21:110. doi: 10.1186/s13059-020-02030-2 (PMC7212609; doi:10.1186/s13059-020-02030-2)
Supplement: Supplementary file 1 — Additional file 1: Supplementary tables. Table S1. Dataset used in this study. Table S2. Reagents used in this study. Table S3. Software and algorithms used in this study. [file 13059_2020_2030_MOESM1_ESM.docx]

**Additional file 2: Supplementary Tables S1-S4**

**Table S1.** Dataset used in this study.

**Table S2.** Reagents used in this study.

**Table S3.** Software and algorithms used in this study.

**Table S4.**

**Table S1:** Dataset used in this study

| **ChIA-PET** | | | | | | |
| --- | --- | --- | --- | --- | --- | --- |
| Cell | Factor | Treatment | GEO accession | Uniquely mapped PETs | Intra-chr. PETs | Data source |
| PR9 | PML | control | GSE137662 | 10,376,163 | 616,023 | This study |
|  |  | ZnSO4 4h | GSE137662 | 61,255,598 | 5,757,440 |  |
|  | RAR𝛼 | control | GSE137662 | 15,608,754 | 3,084,399 |  |
|  |  | ZnSO4 4h | GSE137662 | 58,597,937 | 5,532,885 |  |
|  | RNAPII | control | GSE137662 | 180,539,278 | 11,194,276 |  |
|  |  | ZnSO4 4h | GSE137662 | 66,568,749 | 65,521,063 |  |
|  | CTCF | control | GSE137662 | 91,515,096 | 6,184,381 |  |
|  |  | ZnSO4 4h | GSE137662 | 36,355,723 | 4,255,073 |  |
| NB4 | PML | NA | GSE137662 | 97,332,648 | 34,880 |  |
|  | RAR𝛼 | NA | GSE137662 | 172,674,019 | 20,169 |  |
|  | RNAPII | NA | GSE137662 | 29,173,744 | 29,832 |  |
|  | CTCF | NA | GSE137662 | 22,674,016 | 58,471 |  |
| **ChIP-Seq** | | | | | | |
| Cell | Factor | Treatment | GEO accession | Uniquely mapped reads | Peak numbers (MACS) | Data source |
| PR9 | PU.1 | control | GSE137662 | 42,939,412 | 7,615 | This study |
|  |  | ZnSO4 4h | GSE137662 | 39,768,820 | 4,251 |  |
|  | CEBPB | control | GSE137662 | 43,253,760 | 3,879 |  |
|  |  | ZnSO4 4h | GSE137662 | 41,530,777 | 2,865 |  |
|  | IRF1 | control | GSE137662 | 45,188,813 | 3,829 |  |
|  |  | ZnSO4 h | GSE137662 | 47,838,743 | 2,456 |  |
|  | P300 | control | GSE137662 | 15,813,190 | 31,622 |  |
|  |  | ZnSO4 4h | GSE137662 | 9,150,658 | 16,479 |  |
|  | H3K9K14ac | control | GSM468215 | 11,261,531 | 30,150 | Martens *et al* ., 2010 |
|  |  | ZnSO4 4h | GSM468216 | 11,251,479 | 32,824 | Martens *et al* ., 2010 |
| **RNA-Seq (ribosomal RNA depleted)** | | | | | | |
| Cell | Experiment | Treatment | GEO accession | Uniquely mapped reads |  | Data source |
| PR9 |  | control | GSE137662 | 108,254,542 |  | This study |
|  |  | ZnSO4 4h | GSE137662 | 108,122,444 |  |  |
|  |  | ZnSO4 6h | GSE137662 | 112,468,120 |  |  |
|  |  | ZnSO4 12h | GSE137662 | 89,297,626 |  |  |
|  |  | ZnSO4 24h | GSE137662 | 107,669,338 |  |  |
|  |  | ZnSO4 4h+ATRA 24h | GSE137662 | 63,868,024 |  |  |
|  |  | ZnSO4 4h+ATRA 48h | GSE137662 | 50,140,652 |  |  |
| NB4 |  | control | GSE137662 | 59,455,644 |  |  |
|  |  | ATRA 24h | GSE137662 | 57,918,501 |  |  |
|  |  | ATRA 48h | GSE137662 | 59,042,779 |  |  |

**Table S2:** Reagents used in this study

| Resource | Source | Identifier | Experiments or analysis |
| --- | --- | --- | --- |
| PML antibody | Santa Cruz | Cat# sc-966X | ChIA-PET |
| RARα antibody | Santa Cruz | Cat# sc-551X | ChIA-PET |
| RNA Polymerase II antibody | Biolegend | Cat# 664912 | ChIA-PET |
| CTCF antibody | Abcam | Cat# ab70303 | ChIA-PET |
| P300 antibody | Abcam | Cat# ab14984 | ChIP-Seq |
| PU.1 antibody | Santa Cruz | Cat# sc-352X | ChIP-Seq |
| CEBPB antibody | Santa Cruz | Cat# sc-150X | ChIP-Seq |
| IRF1 antibody | Santa Cruz | Cat# sc-497X | ChIP-Seq |
| Nextera DNA sample preparation kit | Illumina, Inc | Cat# FC-121-1030 | ChIA-PET |
| Nextera Index kit | Illumina, Inc | Cat# FC-121-1011 | ChIA-PET |
| ScriptSeq RNA-seq library preparation kit | Illumina, Inc | Cat# SSV21124 | RNA-seq |
| Ribo-Zero rRNA Removal Kit (Human/Mouse/Rat) | Illumina, Inc | Cat# MRZH11124 | RNA-seq |
| TruSeq ChIP library preparation kit -Set A | Illumina, Inc | Cat# IP-202-1012 | ChIP-Seq |
| LightCycler 480 SYBR Green I Master | Roche | Cat# 04887352001 | ChIP-Seq, ChIA-PET |
| DNA Clean & Concentrator-5 | Zymo research | Cat# 4013 | ChIA-PET |
| QIAprep Spin Miniprep Kit | Qiagen | Cat# 27106 | ChIA-PET, ChIP-seq |
| RNeasy Mini Kit | Qiagen | Cat# 74106 | RNA-seq |
| ZnSO4⋅7H2O | Sigma | Cat# Z0251 | Cell culture |
| All-trans retinoic acid (ATRA) | Sigma | Cat# R2625 | Cell culture |
| EGS (ethylene glycol bis(succinimidyl succinate)) | ThermoFisher | Cat# 21565 | ChIA-PET |
| Formaldehyde | Millipore | Cat# 104003 | ChIA-PET, ChIP-seq |
| Customized FISH probe | MYcroarray | NA | 3D-DNA FISH |
| T4 DNA polymerase | Promega | Cat# 4421 | ChIA-PET |
| Klenow Fragment (3’-5’ Exo) | NEB | Cat# M0212M | ChIA-PET |
| T4 DNA Ligase, HC (30 U/µL) | ThermoFisher | Cat# EL0013 | ChIA-PET |
| T4 DNA Ligase Buffer | ThermoFisher | Cat# 46300018 | ChIA-PET |
| T4 DNA Ligase Reaction Buffer | NEB | Cat# B0202S | ChIA-PET |
| DNA Polymerase I (*E. coli*) | NEB | Cat# M0209L | ChIA-PET |
| Dynabeads™ Protein G | ThermoFisher | Cat# 10009D | ChIA-PET |
| AMPURE XP beads | Beckman Coulter | Cat# A63881 | ChIA-PET |
| Dynabeads™ M-280 Streptavidin | ThermoFisher | Cat# 11206D | ChIA-PET, ChIP-seq |
| 2% Agarose Gel Cassette | Sage Science | Cat# BDF2010 | ChIA-PET |
| ProLong™ Gold Antifade Mountant with DAPI | ThermoFisher | Cat# P36931 | 3D-DNA FISH |
| MmeI | NEB | Cat# R0637l | ChIA-PET |
| Proteinase K | ThermoFisher | Cat# 25530049 | ChIA-PET, ChIP-Seq |
| S-adenosylmethionine (SAM) | NEB | Cat# B9003S | ChIA-PET |
| GlycoBlue™ Coprecipitant | ThermoFisher | Cat# AM9516 | ChIA-PET |
| Sodium Acetate (3 M), pH 5.5, RNase-free | ThermoFisher | Cat# AM9740 | ChIA-PET |
| 2% Agarose Gel Cassettes | Sage Science | Cat# BDF2010 | ChIA-PET |

**Table S3:** Software and algorithms used in this study

| Resource (Reference) | Identifier |
| --- | --- |
| Bowtie2[1] | <http://bowtie-bio.sourceforge.net/index.shtml> |
| Picard (Broad Institute) | <http://broadinstitute.github.io/picard/> |
| Edge R[2] | https://bioconductor.org/packages/release/bioc/html/edgeR.html |
| MACS[3] | <https://github.com/taoliu/MACS> |
| 3D-GNOME[4] | <http://nucleus3d.cent.uw.edu.pl/> |
| GREAT[5] | <http://great.stanford.edu/public/html/> |
| IMARIS 9 (BITPLANE) | http://www.bitplane.com/releasenotes/imaris930.aspx |
| Juicer/Juicebox[6,7] | <http://aidenlab.org/juicebox/> |
| JASPAR[8] | <http://jaspar.genereg.net> |
| ROSE[9,10] | [http://younglab.wi.mit](http://younglab.wi.mit.edu/super%20_enhancer_code.html).edu/super_enhancer_code.html |

**Table S4.** Differential expression of genes (267) affected by PML-RARα in PR9 cells (in Excel)
