# Supplementary material for: Chromatin topology reorganization and transcription repression by PML-RARα in acute promyeloid leukemia
Source: Genome Biol. 2020 May 11;21:110. doi: 10.1186/s13059-020-02030-2 (PMC7212609; doi:10.1186/s13059-020-02030-2)
Supplement: Supplementary file 2 — Additional file 2: Supplementary figures. Figure S1. Comparison of CTCF and RNAPII binding and looping in PR9 and PR9+Zn cells. Figure S2. PML-RARα-mediated chromatin interaction domain and impacts on the myeloid genome. Figure S3. Transcription repression of myeloid specific genes by PML-RARα. Figure S4. Further details of motif analysis around PML-RARα binding sites. Figure S5. Super-enhancers of myeloid specific genes affected by PML-RARα. Figure S6. Additional examples of PML-RARα in NB4 cell. [file 13059_2020_2030_MOESM2_ESM.docx]

**Additional file 1: Supplementary Figures S1-S6**

**Figure S1** Comparison of CTCF and RNAPII binding and looping in PR9 and PR9+Zn cells.

**Figure S2** PML-RARα-mediated chromatin interaction domain and impacts on the myeloid genome.

**Figure S3** Transcription repression of myeloid specific genes by PML-RARα.

**Figure S4** Further details of motif analysis around PML-RARα binding sites.

**Figure S5** Super-enhancers of myeloid specific genes affected by PML-RARα.

**Figure S6** Additional examples of PML-RARα in NB4 cell.


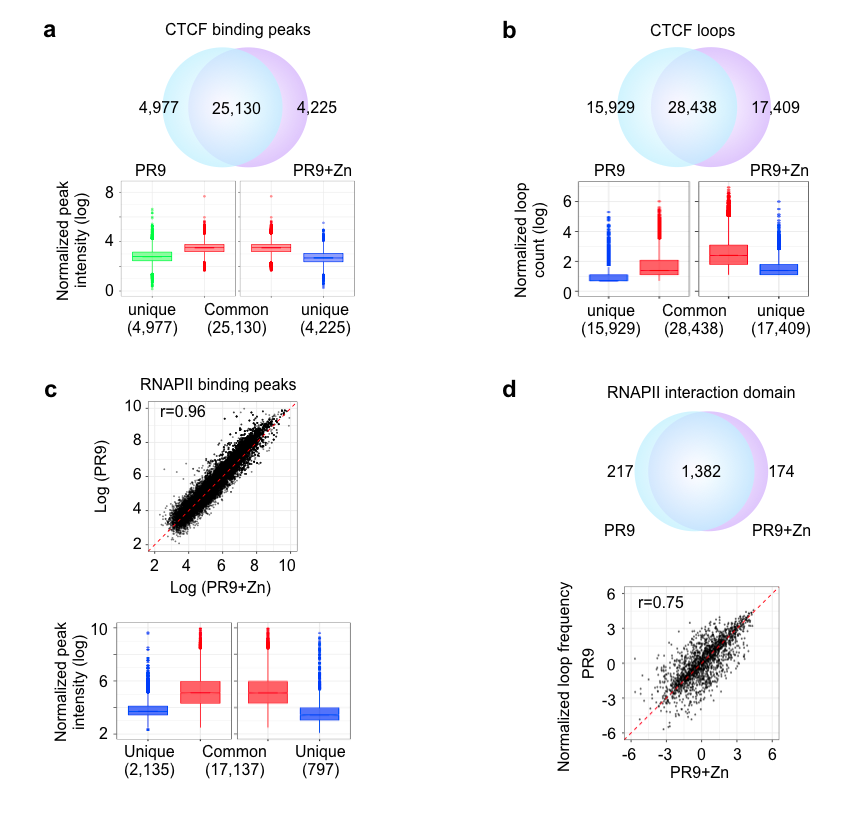


**Figure S1**

**Figure S1. Comparison of CTCF and RNAPII binding and looping in PR9 and PR9+Zn cells**. Related to Figure 1. **a-b.** CTCF binding peaks (a) and loops (b). Venn diagram shows common and unique CTCF peaks or loops between PR9 and PR9+Zn cells. Box plots show the intensities of peaks or loops that were unique or common to PR9 and PR9+Zn cells. **c.** Scatter plot of RNAPII binding peaks in PR9 and PR9+Zn cells (top). Box plots show the intensities of peaks that were unique or common to PR9 and PR9+Zn cells (bottom). **d.** Venn diagram shows RNAPII chromatin interaction domains between PR9 and PR9+Zn cells (top). Scatter plot displays the correlation of RNAPII looping frequency in PR9 and PR9+Zn cells (bottom).

**
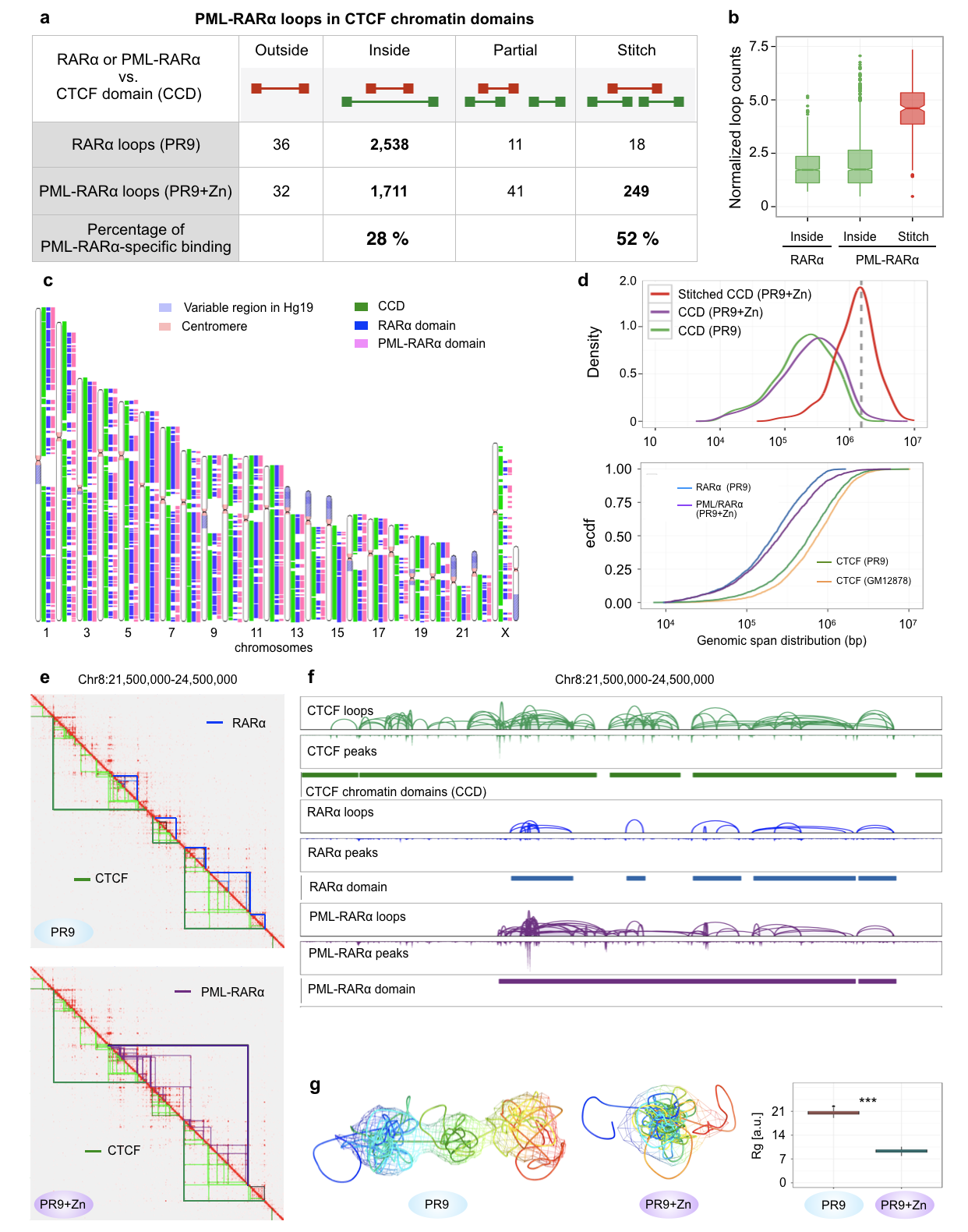
**

**Figure S2**

**Figure S2. PML-RARα-mediated chromatin interaction domain and impacts on the myeloid genome.** Related to Figure 2. **a.** Categorization of RARα in PR9 and PML-RARα in PR9+Zn cells in relation to CTCF-defined chromatin domains. **b.** Boxplot shows normalized chromatin contact frequency for RARα inside CCD domain in PR9 cells, and for PML-RARα inside CCD domain, as well as CCD domains stitched together in PR9+Zn cells. Kolmogorov-Smirnov test (K-S test) was used to test difference. ** for p < 0.001; n.s. for not significant (p > 0.001). **c.** Whole-genome view shows the distribution of CCD, RARα and PML-RARα domains in each chromosome. **d.** Size distribution CCD and “stitched” CCD in PR9 and PR9+Zn cells (top). The ECDF plot of chromatin loops for RARα, PML-RARα, and CTCF in PR9 and PR9+Zn cells, with the CTCF loops in GM12878 as a reference (bottom). **e.** Integrated 2D chromatin contact maps for a 3-Mb genomic segment from the combined (red) CTCF, RNAPII, and RARα ChIA-PET data in PR9 cells, as well as CTCF, RNAPII, and PML-RARα ChIA-PET data in PR9+Zn cells. Light green and dark green triangles indicate CTCF loop and CCD, respectively; light blue and dark blue triangles indicate RARα loops and domains in PR9 cells; and the light and dark purple triangles depict PML-RARα loops and domains in PR9 cells. **f.** Browser views of chromatin interaction loop, peaks, and chromatin domain of CTCF (green), RARα (blue), and PML-RARα (purple). **g.** 3D chromatin folding modeling for the genomic segment (same as in E and F): simulated average structure and ensemble in PR9 (left) and in cells (middle). The boxplot shows radial diameter distributions of simulated 3D structures from 300 nuclei examined in PR9 and PR9+Zn cells. K-S test was used to test differences. ** *p* < 2.2e-16.


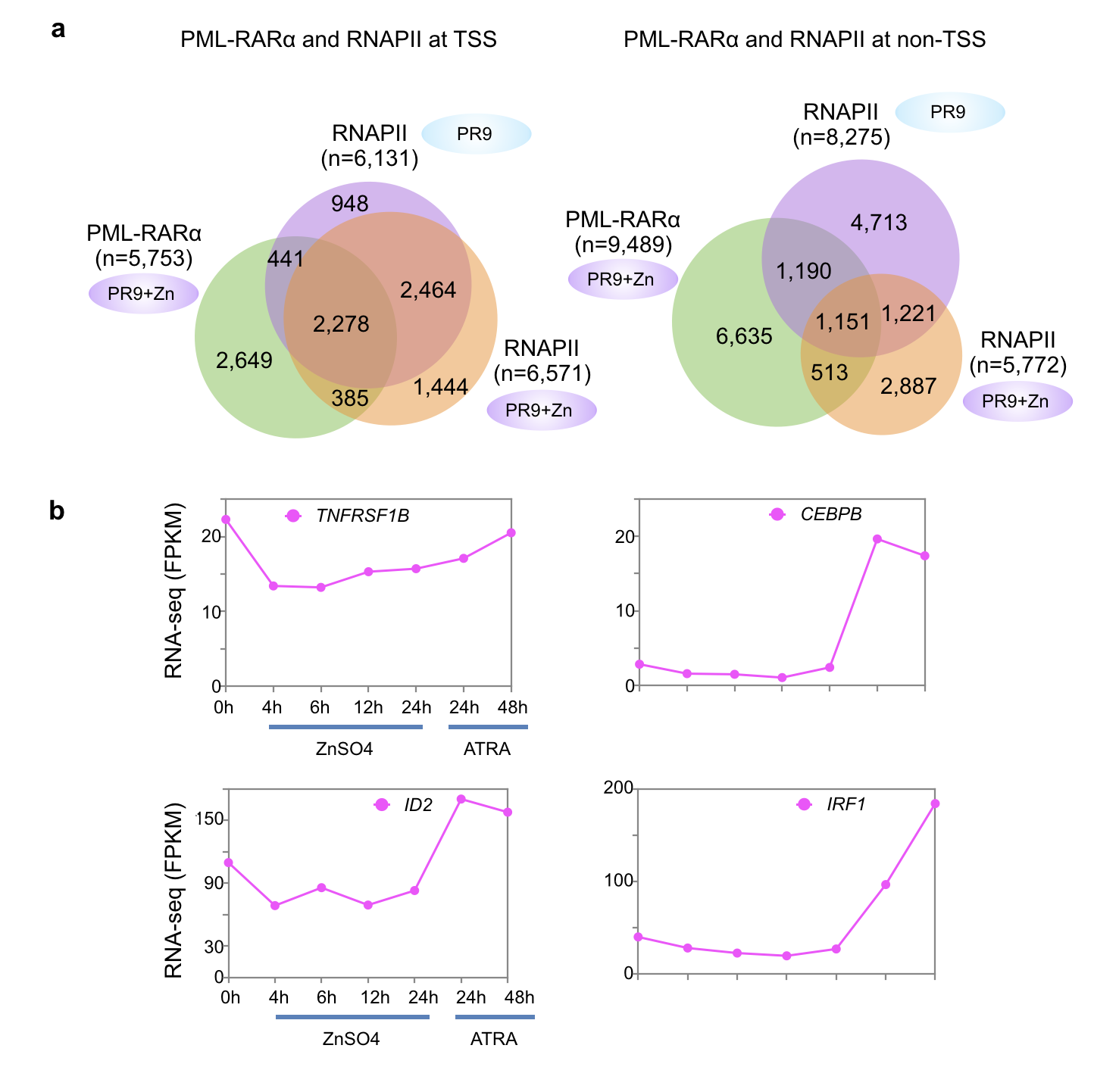


**Figure S3**

**Figure S3. Transcription repression of myeloid specific genes by PML-RARα.** Related to Figure 3. **a.** Venn diagrams show the overlap of binding sites by PML-RARα and RNAPII proximal to TSS of genes (left) and distal to TSS located at non-TSS loci (right). **b.** Line plots show gene (*IRF1*, *CEBPB*, *ID2*, *TNFRSF1B*) expression changes over the time course of ZnSO4 induction for PML-RARα activation and ATRA treatments in in PR9 cells.


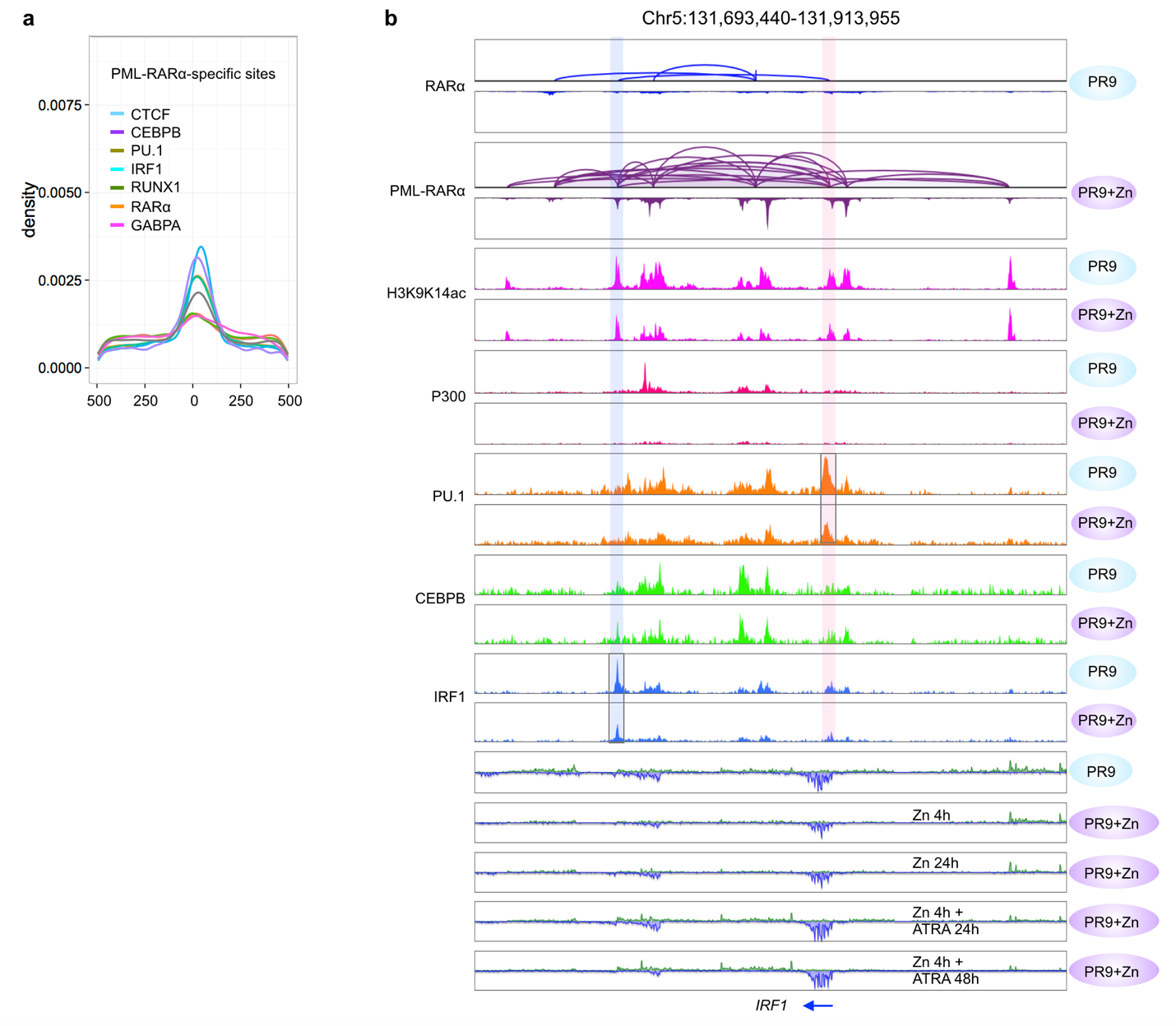


**Figure S4**

**Figure S4. Further details of motif analysis around PML-RARα binding sites.** Related to Figure 4. **a.** DNA binding motifs enriched (CTCF, CEBPB, PU.1, IRF1, RUNX1, RARA, and GABPA) at PML-RARα binding sites. **b.** An example browser view of chromatin interaction loops and binding peaks for RARα and PML-RARα,from ChIA-PET data, and the peaks from ChIP-seq data of H3K9K14ac and specific TFs at *IRF1* loci in both PR9 and PR9+Zn cells. The expression data (RPKM) for *IRF1* expression are given in each RNA-seq track, *P* value = 8.90e-20 (control versus Zn_4h).


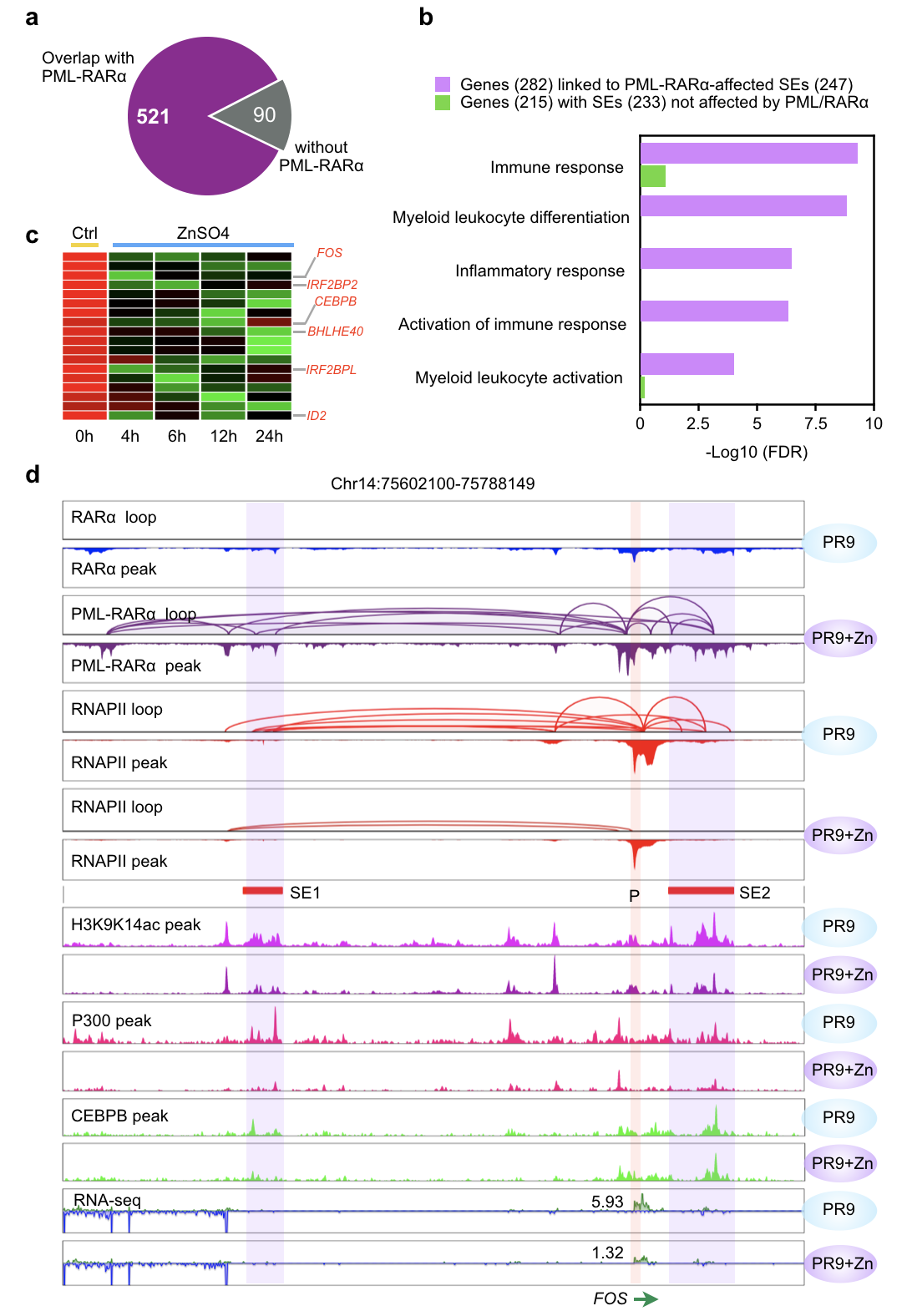


**Figure S5**

**Figure S5. Super-enhancers of myeloid specific genes affected by PML-RARα.** Related to Figure 5. **a.** Pie chart of SEs overlapped with PML-RARα anchors. **b.** GO enrichment analysis of genes (n=247) associated with SEs that were affected by PML-RARα, and for the genes associated with SEs that were not affected by PML-RARα. **c.** Heatmap shows expression profiles of PML-RARα-affected genes that are critical for myeloid development. **d.** An example of two SEs at their target gene *FOS* locus with tracks of PML-RARα and RNAPII ChIA-PET data (loops and peaks), along with ChIP-seq data of H3K9K14ac, P300 and CEBPB, as well as RNA-seq data from both PR9 and PR9+Zn cells. The locations of the promoter and the two SEs are highlighted. The expression data (RPKM) for *FOS* expression are given in each RNA-seq track, *P* value = 6.38e-3 (control versus Zn_4h).

**
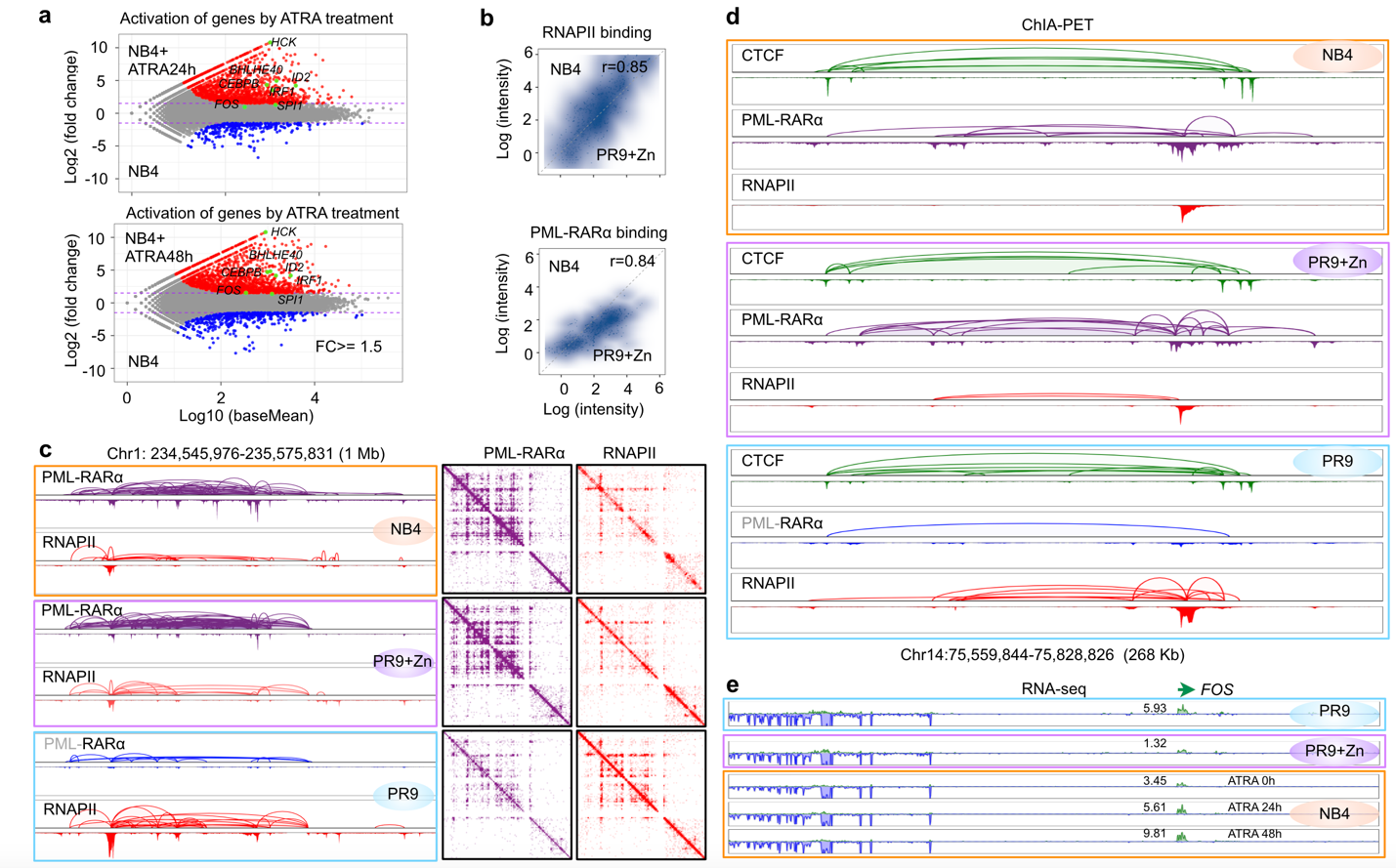
**

**Figure S6**

**Figure S6. Additional examples of PML-RARα in NB4 cell.** Related to Figure 6. **a.** Scatter plots show differentially expressed genes in NB4 cells and NB4 cells with ATRA treatment for 24 hours (top) and 48 hours (bottom). Red dots highlight genes activated by ATRA treatment, with representative genes HCK, BHLHE40, CEBPB, IRF1, FOS, and SPI1. **b.** Contour plots for correlations of RNAPII binding (up) and PML-RARα binding (low) intensity at the 119 target genes between NB4 vs. PR9+Zn cells. Correlation coefficient r-values are provided. **c.** Example shows comparable chromatin interaction profiles by PML-RARα and RNAPII in NB4 cells and PR9+Zn cells, whereas in PR9 cells, the RNAPII interaction data were much stronger. Left, browser view of loops and peaks. Right, 2D contact maps. **d.** Another example shows the local chromatin loops and peaks by CTCF (green), PML-RARα (purple) or RARα (blue), and RNAPII (red) at the *FOS* locus in NB4 cells (orange box), PR9+Zn cells (purple box), and PR9 cells (in blue box). **e.** *FOS* gene expression in PR9 and PR9+Zn cells, and in NB4 cells with ATRA treatment for 24 and 48 hours.
