# Supplementary material for: Chromatin topology reorganization and transcription repression by PML-RARα in acute promyeloid leukemia
Source: Genome Biol. 2020 May 11;21:110. doi: 10.1186/s13059-020-02030-2 (PMC7212609; doi:10.1186/s13059-020-02030-2)
Supplement: Supplementary file 3 — Additional file 3. Supplementary methods. 1. ChIA-PET data processing and clustering of interaction PETs. 2. Comparison of ChIA-PET data for calling of binding peaks, loops, and chromatin domains. 3. Identification and classification of RARα and PML-RARα binding sites. 4. Identification and characterization of PML-RARα-mediated chromatin interactions. 5. Visualization of 2D contact map and BASIC browser. 6. 3D chromatin structure modeling, measurement of 3D structure condensation and visualization. 7. Identification of binding motifs at PML-RARα peak regions. 8. Comparison of RNAPII-mediated chromatin interactions between control and treatment condition. 9. RNA-Seq and differential gene expression analysis. 10. ChIP-Seq analysis. 11. Identification and characterization of super-enhancers. 12. Quantification and statistical analysis methods. [file 13059_2020_2030_MOESM3_ESM.docx]

**Additional file 3: Supplementary methods**

1. ChIA-PET data processing and clustering of interaction PETs.
2. Comparison of ChIA-PET data for calling of binding peaks, loops, and chromatin domains.
3. Identification and classification of RARα and PML-RARα binding sites.
4. Identification and characterization of PML-RARα-mediated chromatin interactions.
5. Visualization of 2D contact map and BASIC browser.
6. 3D structure modeling, measurement of 3D structure condensation and visualization.
7. Identification of binding motifs at PML-RARα peak regions.
8. Comparison of RNAPII-mediated chromatin interactions between control and treatment condition.
9. RNA-Seq and differential gene expression analysis.
10. ChIP-Seq analysis.
11. Identification and characterization of super-enhancers.
12. Quantification and statistical analysis methods.
13. **ChIA-PET data processing and clustering of interaction PETs**

In this study, ChIA-PET data was produced by the long-reads ChIA-PET and short-reads ChIA-PET protocols. The long-read ChIA-PET and short-read ChIA-PET data sets were processed using an updated ChIA-PET data processing pipeline, called ChIA-PIPE [11]. From each library data, protein binding peaks, chromatin loops and chromatin interaction domains were identified.

1. **Comparison of ChIA-PET data for calling of binding peaks, loops, and chromatin domains**

To compare the CTCF ChIA-PET data, we intersected the CTCF peak regions between the PR9 and PR9+Zn data, and identify unique and common sets of the peaks (Supplementary Fig. 1d). For each condition, we sampled the same number of PET to calculate CTCF binding intensity at each peak region (Supplementary Fig. 1a). To compare the loops, we extended ±1.5 kb to each anchor of CTCF loops to intersect common loops. Loop showing both overlapped anchors with that of a loop from different datasets was considered as a common loop (Supplementary Fig. 1b).

Considering that the CTCF ChIA-PET data showed the high similarity between PR9 and PR9+Zn cells, we merged the two datasets together, and used the combined data to generate a reference 3D genome map mediated by CTCF for myeloid cell PR9, which includes CTCF peaks, CTCF loops, CTCF chromatin domains (CCD) similar as we did for the 3D genome map of GM12878 (Tang et al., 2015). The same approach was also applied to characterize RARα, RNAPII and PML ChIA-PET data generated from PR9 and PR9+Zn cells. The results were shown in Fig. 2a.

1. **Identification and classification of RAR**α **and PML-RAR**α **binding sites**

We generated PML and RARα ChIA-PET libraries for PR9 and PR9+Zn cells, respectively. Using ChIA-PIPE, we called peaks and loops for corresponding factors. As expected, no significant signals for peaks and loops were detected PML ChIA-PET data from PR9 cells, because PML is not a chromatin protein. The binding peaks detected in the RARα ChIA-PET data in PR9 represented the endogenous RARα binding sites. In PR9+Zn cells, the binding peaks detected by RARα ChIA-PET data could for both of the endogenous RARα and the induced PML-RARα protein, while the binding peaks identified by PML ChIA-PET data should only represent the induced PML-RARα fusion protein. To identify high-confidence data, we considered only the peak regions detected more than two datasets in these three ChIA-PET experiments in PR9 and PR9+Zn cells as illustrated in Fig. 2b. According to binding patterns, the peak regions were classified into three categories (Fig. 2b): endogenous RARα-specific peaks (Type I), shared peaks by endogenous RARα and the induced PML-RARα (Type II), and PML-RARα-specific peaks (Type III). In type II category, the endogenous RARα and the induced PML-RARα were co-occupied at the same genomic regions.

1. **Identification and characterization of PML-RAR**α**-mediated chromatin interactions**

The PML- and RARα- ChIA-PET datasets essentially represent the detection of the fusion protein PML-RARα. To comprehensively analyze the chromatin interactions mediated by PML-RARα, we merged two datasets together. The combined PETs were used to identify high-confidence PML-RARα loops and domain similar as we did for CTCF (Fig. 2a).

1. **Visualization of 2D contact map and BASIC browser**

The pairwise contact data from short-read ChIA-PET and long-read ChIA-PET were combined. We produced 2D contact map files (hic files) using Juicer tools [7] (version 1.6.1) (<https://github.com/aidenlab/juicer/wiki/Download>), and visualized them using Juicebox (version 1.6.1) [6]. Ten different resolutions were applied from 2.5Mb to 10Kb. We chose none normalization in Juicebox for our ChIA-PET 2D contact map visualization. We generated contact map files for individual single-factor conditions, CTCF, RNAPII, PML-RARα, and RARα for control and treatment cases, respectively. In ChIA-PIPE, there is a BASIC browser viewer for visualizing loops and peaks for each ChIA-PET dataset.

1. **3D structure modeling, measurement of 3D structure condensation and visualization**

The modeling was performed using 3D-GNOME software we developed earlier [4]. The models were generated using a combined CTCF, RNAPII, and PML-RARα ChIA-PET loop dataset for control and treatment cases, respectively. The PET count threshold was set to 3 for CTCF and PML-RARα loops and 2 for RNAPII loops. Loops, which PET count is less than the threshold, were filtered out. Also, we took the loops, where both loop anchors are inside of the target genomic region, as an input for 3D-GNOME. Neither anchor or sub anchor heatmaps nor CTCF motifs orientations optional energy terms were used in the modeling. An ensemble of 100 structures was created for each target genomic region. The 3D polymer models from 3D-GNOME are based on chromatin loops, and thus the genomic distances between consecutive beads in each model are variable. To assure uniform bead coverage over the target genomic region, the 3D model structures were interpolated using a cubic spline method with a 1Kb interval. 3D visualizations (Fig. 2h and Supplementary Fig. 2g) were created using the UCSF Chimera package. In every panel, the medoid structure (i.e. a structure for which the average pairwise dissimilarity to all the other structures is minimal; medoid can be intuitively thought of as a central or average structure) is presented, with the color corresponding to the genomic position. Additionally, an ensemble cloud that represents the average conformation of the whole ensemble of structures is shown. The cloud was calculated using the Calculate Occupancy tool in Chimera (the structures were previously co-aligned). While the locations of the individual loops varied between individual models, the ensemble clouds demonstrate that the overall shapes of 100 model structures were homogenous. Generally, the control structures consist of multiple, well-separated domains connected by linker regions, whereas the treatment structures are compacted to a single globule. To compare the level of compaction between control and treatment structures, we calculated the radius of gyration for individual structures (Fig. 2h and Supplementary Fig. 2g).

1. **Identification of binding motifs at PML-RAR**α **peak regions**

The DNA sequences in PML-RARα binding peaks were extracted for scanning protein binding consensus sequences. The position weight matrix for RARα, PU.1, IRF1, CEBPB, RUNX1, GABPA and CTCF motifs were downloaded from the JASPAR database (<http://jaspar.genereg.net>) [8]. FIMO from MEME suite (version 4.12) was applied to identify of consensus sequences for each protein factor. The threshold of p-value 10^-4^ was applied to filter output of FIMO. The occurrence frequency of consensus sequence for each protein factor was plotted surrounding RARα consensus sequence in a 1kb window (Fig. 4a and Supplementary Fig. 4a). The plot shows consensus sequences of transcription factors, including PU.1, IRF1 and CEBPB, are proximal to the consensus sequences of RARα in PML-RARα binding regions, which suggests PML-RARα may occupy same regions with transcription factors.

1. **Comparison of RNAPII-mediated chromatin interactions between control and treatment condition**

We described the procedures of identifying RNAPII chromatin interaction domains with significant changes in RNAPII-mediated chromatin interactions. We first called the union regions of joint RNAPII chromatin interaction domains from control and treatment. We then calculated numbers of inter-ligation PETs in the union regions at control and treatment, respectively. Similarly, the accumulated PET counts of PET clusters in the union regions were also calculated at control and treatment, respectively. We treated control and treatment conditions as the two groups, and inter-ligation PETs and clustered PETs as the two categories, generating a 2 X 2 contingency table as shown below for Chi-Square test. The R package chisq.test was applied to calculate the p-value. The RNAPII chromatin contact domain was considered with a significant change in chromatin interaction when p-value less than 0.01.

| **Group** | **Categories** | |
| --- | --- | --- |
|  | **inter-ligation PETs** | **clustered PETs from loops** |
| Control | N_a_ | N_b_ |
| Treatment | M_a_ | M_b_ |

Statistical numbers of RNAPII involved loop difference between PR9 cells and ZnSO4 treated PR9 cells at the local regions were summarized in Supplementary Fig. 4b, and *p*-values were estimated from two-side Chi-square test.

1. **RNA-Seq and differential gene expression analysis**

Sequenced reads were mapped to the human reference genome hg19 using Bowtie2 (<http://bowtie-bio.sourceforge.net/bowtie2/index.shtml>) [1] with default parameters. Only uniquely mapped reads were detained for the downstream analysis. The counts of mapped reads fell in exons of each gene were calculated and were then applied to identify differentially expressed genes using R package ‘edge R’ [2]. The expression values for each gene was normalized using reads per kilobase per million (RPKM) mapped reads. The expression heat maps were generated with RPKM values of genes using R package ‘heatmap2’ (Fig. 3b). The log fold-change of 1 and *p*-value of 0.01 were applied to identify differentially expressed gene between control and treatment conditions in PR9 cells or between control and treatment conditions in NB4 cells. The gene ontology enrichment analysis was performed for differentially expressed genes using GREAT (<http://great.stanford.edu/public/html/>) (Fig. 3c). To compare the similarity in gene expression between PR9 and NB4 cells in a variety of conditions, genes with RPKM >= 1 in both PR9 and NB4 cells were applied to calculate Pearson correlation using R package stats (Fig. 6b).

1. **ChIP-Seq analysis**

Sequenced reads were mapped to the human reference genome hg19 using bwa-mem (version 0.7.16) with default parameters. Only uniquely mapped reads were detained for the downstream analysis. In order to compare the ChIP-Seq profiles between control and treatment, we sampled the same number of mapped reads for peak calling in both control and treatment conditions. MACS 1.4.2 was used for identification of peaks using default parameters. If genomic distance between peaks is less than 2 kb, the peaks were merged together and applied for downstream analysis. To compare the correlation of RNAPII and PML-RARα binding profiles between PR9 and NB4 cells, the values of reads per million (RPM) for peak regions were applied to calculate Pearson correlation using R package stats (Fig. 6c).

1. **Identification and characterization of super-enhancers**

We used ROSE (<http://younglab.wi.mit.edu/super_enhancer_code.html>) [9,10] with default setting to discovery super-enhancer based on genome-wide H3K9K14ac profile. Super-enhancers were identified as regions with slope denoting (focus ratio)/(region size annotation enhancer) greater than 1. The super-enhancer regions were identified in control and treatment condition, respectively (Fig. 5b). The super-enhancer regions overlapped more than 70% between conditions were categorized as common super-enhancers. Except the common super-enhancers, others were grouped as condition-specific super-enhancers. The categorized super-enhancer regions were intersected with PML-RARα binding peaks, and only super-enhancer regions overlapped with PML-RARα binding peaks were detained for further characterization (Supplementary Fig. 6a, Fig. 6c).

1. **Quantification and statistical analysis methods**

Kolmogorov-Smirnov test (K-S test) was applied in Figure 2h, Supplementary Figure 2b, Supplementary Figure 2g and Figure 5d. Mann-Whitney u test was applied in Figure 2g. All statistical tests used two-side tests.

**References Cited in Supplementary Information**

1. Langmead B, Trapnell C, Pop M, Salzberg SL. Ultrafast and memory-efficient alignment of short DNA sequences to the human genome. Genome Biol. 2009;10:R25.

2. Robinson MD, McCarthy DJ, Smyth GK. edgeR: a Bioconductor package for differential expression analysis of digital gene expression data. Bioinformatics. 2010;26:139–40.

3. Zhang Y, Liu T, Meyer CA, Eeckhoute J, Johnson DS, Bernstein BE, et al. Model-based Analysis of ChIP-Seq (MACS). Genome Biol. 2008;9:R137.

4. Szalaj P, Michalski PJ, Wróblewski P, Tang Z, Kadlof M, Mazzocco G, et al. 3D-GNOME: an integrated web service for structural modeling of the 3D genome. Nucleic Acids Res. 2016;44:W288-293.

5. McLean CY, Bristor D, Hiller M, Clarke SL, Schaar BT, Lowe CB, et al. GREAT improves functional interpretation of cis-regulatory regions. Nat Biotechnol. 2010;28:495–501.

6. Durand NC, Robinson JT, Shamim MS, Machol I, Mesirov JP, Lander ES, et al. Juicebox Provides a Visualization System for Hi-C Contact Maps with Unlimited Zoom. Cell Syst. 2016;3:99–101.

7. Durand NC, Shamim MS, Machol I, Rao SSP, Huntley MH, Lander ES, et al. Juicer Provides a One-Click System for Analyzing Loop-Resolution Hi-C Experiments. Cell Syst. 2016;3:95–8.

8. Khan A, Fornes O, Stigliani A, Gheorghe M, Castro-Mondragon JA, van der Lee R, et al. JASPAR 2018: update of the open-access database of transcription factor binding profiles and its web framework. Nucleic Acids Res. 2018;46:D260–6.

9. Lovén J, Hoke HA, Lin CY, Lau A, Orlando DA, Vakoc CR, et al. Selective inhibition of tumor oncogenes by disruption of super-enhancers. Cell. 2013;153:320–34.

10. Whyte WA, Orlando DA, Hnisz D, Abraham BJ, Lin CY, Kagey MH, et al. Master transcription factors and mediator establish super-enhancers at key cell identity genes. Cell. 2013;153:307–19.

11. Capurso D, Wang J, Tian SZ, Cai L, Namburi S, Lee B, et al. ChIA-PIPE: A fully automated pipeline for ChIA-PET data analysis and visualization [Internet]. Bioinformatics; 2018 Dec. Available from: http://biorxiv.org/lookup/doi/10.1101/506683
