# Supplementary material for: Chromatin topology reorganization and transcription repression by PML-RARα in acute promyeloid leukemia
Source: Genome Biol. 2020 May 11;21:110. doi: 10.1186/s13059-020-02030-2 (PMC7212609; doi:10.1186/s13059-020-02030-2)
Supplement: Supplementary file 5 — Additional file 5. Review history. [file 13059_2020_2030_MOESM5_ESM.docx]

Review History

**First round of review**

**Reviewer 1**

**Are you able to assess all statistics in the manuscript, including the appropriateness of statistical tests used?**

Yes, and I have assessed the statistics in my report.

**Comments to author:**

The simultaneous presence of histone acetyltransferases and HDACs is in accordance with the emerging picture of a balanced interplay of the antagonistic activities of histone-modifying enzymes, such as histone acetyltransferases and HDACs or methyltransferases and demethylases.  The dynamic balance of histone acetylation levels at chromatin regions was known to be maintained by the activity of histone acetyltransferases/HDACs as well as histone demethylase (such as LSD1-containing complexes). Recent studies suggested PML-RARα, AML1-ETO and other oncofusion proteins could tip the balance by stabilizing deacetylase activity at their target regions in leukemic cells. In this study, the authors conducted a comprehensive investigation of the corresponding chromatin topological dynamics and transcription repression of differentiation targets by PML-RARα in APL in a nice inducible and rescue system pair and demonstrated consistency with that in APL patient-derived NB4 cells. As stated by the authors:" Intriguingly, our data indicated that PML/RAR did not directly interfere with CTCF binding and chromatin looping, but rather that many of the PML/RAR-mediated chromatin loops overlapped the boundaries of CTCF-defined topological structures, and acted as a "stitch" or "staple" to interconnect separate chromatin topological structures into much larger domains with more condensed configuration, thereby reshaping the chromatin topology in normal myeloid cells leading to leukemogenesis." Competing with P300 presumably would also require open accessible regions dynamically, which would be against naïve notion of "compactification", I suggest the authors could offer more explanation or insight on this point. Another question is if the authors also found other important genome variations related to the 3D genome alternation (e.g. translocations in NB4 cells, other than ectopically expressed oncofusion gene)? Since PML-RAR is dominant genitive, it would be helpful if the authors could indicate how future 3D Genome study could reveal how oncofusion/translocation was created in the first place? Overall this is a very interesting study, which should positively impact the cancer field.
Few potential typos should be carefully examined and fixed: e.g.  "dereived" should be "derived" on line 90, "PR9+Zn" should be "PR9"?...Also suggest to use PML-RAR instead of PML/RAR.

**Reviewer 2**

**Are you able to assess all statistics in the manuscript, including the appropriateness of statistical tests used?**

There are no statistics in the manuscript.

**Comments to author:**

The goal of this project is to study whether the APL-specific fusion protein PML/RARa contribute to the 3D genome structure and APL-specific gene expression.  The experiment design is very solid by using a well-established APL cell line (PR9). When treated with ZnsO4, the RP9 cells will express high level of the fusion protein PML/RARa. Further, the authors also performed rescue experiments - treating the PR9+ZnsO4 cells with the APL drug (ATRA), and further examine whether the cells will reassemble the naïve state. For validation purpose, they also performed the similar set of experiments in in patient-derived APL cells, which can mimic PR9+ZnsO4 cells.

I think their major conclusion is that the scaffold of the human genome are still CTCF-mediated loops/loops domains. The function of PML/RARa in 3D is that they add more local loops (Figure 1B and 1C) and further affect the local domains. Overall, the figures are very clean and the writing is clear. This is one of those rare situations that as a reviewer, I don't have much suggestions to give, because the authors have done all the essential experiments that I can think of. The only thing that coould potentially increase the impact of this work is to check their results in patient samples rather than cell lines, but I understand that patient samples are not easy to get access to and potentially don't add values to our understanding of the function of PML/RARa - so if they can add patient samples, that's superb. If not, I am ok with it too.

At the same time, the most important thing for me is that I am hoping the authors to clarify is the relationship between CTCF/cohesin and PML/RAR〈. In specific,
1.     When treated by ZnsO4 and further followed by treatment with ATRA, how is the expression of CTCF and cohesion complex;
2.     Clearly delineate CTCF loops from PML/RARa loops. Out of all the PML/RARa loops, how many of them overlap with CTCF loops? Can authors discuss whether PML//RARa can block CTCF-mediated loop extrusion?
3.     Similar to the above comment, I really hope the authors can discuss whether non-CTCF-mediated loops (such as PML/RARa, YY1, or ZNF 143) are more transient than CTCF loops. I feel this discussion will be important for this field.
4.     Minor: regarding differential gene expression, the authors need to give significance (such as DESeq). It seems that right now they only rely on RPKM counts.
One last comment, I really appreciate that the authors have released their data on GEO pre-publication. This is rare and I applauded the authors for the effort to keep their research transparent.

**Authors Response**

**Point-by-point responses to the reviewers’ comments:**

Reviewer #1 ===

The simultaneous presence of histone acetyltransferases and HDACs is in accordance with the emerging picture of a balanced interplay of the antagonistic activities of histone-modifying enzymes, such as histone acetyltransferases and HDACs or methyltransferases and demethylases. The dynamic balance of histone acetylation levels at chromatin regions was known to be maintained by the activity of histone acetyltransferases/HDACs as well as histone demethylase (such as LSD1-containing complexes). Recent studies suggested PML-RARα, AML1-ETO and other oncofusion proteins could tip the balance by stabilizing deacetylase activity at their target regions in leukemic cells. In this study, the authors conducted a comprehensive investigation of the corresponding chromatin topological dynamics and transcription repression of differentiation targets by PML-RARα in APL in a nice inducible and rescue system pair and demonstrated consistency with that in APL patient-derived NB4 cells.

*We highly appreciate the reviewer’s positive assessment.*

As stated by the authors:" Intriguingly, our data indicated that PML/RAR did not directly interfere with CTCF binding and chromatin looping, but rather that many of the PML/RAR-mediated chromatin loops overlapped the boundaries of CTCF-defined topological structures, and acted as a "stitch" or "staple" to interconnect separate chromatin topological structures into much larger domains with more condensed configuration, thereby reshaping the chromatin topology in normal myeloid cells leading to leukemogenesis." Competing with P300 presumably would also require open accessible regions dynamically, which would be against naïve notion of "compactification", I suggest the authors could offer more explanation or insight on this point.

*We thank the reviewer for suggesting more explanation about PML-RARα-mediated chromatin compaction. Indeed, we observed that PML-RARα binding and looping lead to the reduction of occupancy by P300 and other factors at corresponding regulatory elements such as promoters and enhancers (Figure 4C and S4b). This observation suggestions that PML-RARα in PR9+Zn cells could potentially compete with P300 and other protein factors at open chromatin loci, potentially condense the chromatin compaction, and thus lead to transcriptional repression. We have added such explanation in page 17 in the revised manuscript.*

Another question is if the authors also found other important genome variations related to the 3D genome alternation (e.g. translocations in NB4 cells, other than ectopically expressed oncofusion gene)?

*This is a very good question. How structural variations and translocations in cancer genomes alter 3D genome organization is becoming more interesting recently, but still largely unknown (Dixon et al., 2018; PMID:30202056). In this study, we focused only on PML-RARα-mediated 3D genome changes.*

Since PML-RAR is dominant genitive, it would be helpful if the authors could indicate how future 3D Genome study could reveal how oncofusion/translocation was created in the first place?

*We thank the reviewer for bring up this important question. Understanding the mechanism that creates oncofusion is critic, but still largely unknown. Although it is believed that oncofusion is a deterministic event and the two parties have to come in close proximity before the translocation taking place, such event itself is likely low frequent (in a few cells in a tissue) and in transient. How to capture that is not an easy task. It is possible to infer the rare events before and after the translocation when comparing the 3D genome mapping data from normal and cancer cells, but when and where it takes place is elusive. In future, advanced 3D genome mapping technologies with single cell and single molecule resolution could be applied to this question and to directly identify the translocation events in individual cells in a large cell population, that would be ideal. We intend to discuss our perspective in a review paper in near future.*

Overall this is a very interesting study, which should positively impact the cancer field. Few potential typos should be carefully examined and fixed: e.g. "dereived" should be "derived" on line 90, "PR9+Zn" should be "PR9"?... Also suggest to use PML-RAR instead of PML/RAR.

*We appreciate the reviewer’s kindness of pointing out the typos. We have now carefully proofread the manuscript, and corrected all previous typos. Yes, we now use PML-RARα in the revised manuscript.*

Reviewer #2: ===

The goal of this project is to study whether the APL-specific fusion protein PML/RARa contribute to the 3D genome structure and APL-specific gene expression. The experiment design is very solid by using a well-established APL cell line (PR9). When treated with ZnsO4, the RP9 cells will express high level of the fusion protein PML/RARa. Further, the authors also performed rescue experiments - treating the PR9+ZnsO4 cells with the APL drug (ATRA), and further examine whether the cells will reassemble the naïve state. For validation purpose, they also performed the similar set of experiments in in patient-derived APL cells, which can mimic PR9+ZnsO4 cells.

I think their major conclusion is that the scaffold of the human genome are still CTCF-mediated loops/loops domains. The function of PML/RARa in 3D is that they add more local loops (Figure 1B and 1C) and further affect the local domains. Overall, the figures are very clean and the writing is clear. This is one of those rare situations that as a reviewer, I don't have much suggestions to give, because the authors have done all the essential experiments that I can think of. The only thing that coould potentially increase the impact of this work is to check their results in patient samples rather than cell lines, but I understand that patient samples are not easy to get access to and potentially don't add values to our understanding of the function of PML/RARa - so if they can add patient samples, that's superb. If not, I am ok with it too.

*We greatly appreciate the reviewer’s positive assessment, and the suggestion to extend our study into clinical patient samples. We agree with the reviewer’s understanding that patient samples are difficult to be obtained, particularly for mechanistic studies. We are now in progress of establishing collaborations with clinician scientists, and hope to uncover more insights of 3D genome organization in leukemogenesis directly from patient samples.*

At the same time, the most important thing for me is that I am hoping the authors to clarify is the relationship between CTCF/cohesin and PML/RAR. In specific,

1. When treated by ZnsO4 and further followed by treatment with ATRA, how is the expression of CTCF and cohesion complex;

*We thank the reviewer for raising a very interesting question. From RNA-seq data, we did not see any significant changes in gene expression of CTCF and RDA21 (cohesin) in all samples. In addition, as shown in Figure 1b and 1e, the CTCF binding and looping in PR9 and PR9+Zn showed no differences, suggesting that the CTCF functions were not affected by the induction of PML-RARα.*

2. Clearly delineate CTCF loops from PML/RARa loops. Out of all the PML/RARa loops, how many of them overlap with CTCF loops? Can authors discuss whether PML//RARa can block CTCF-mediated loop extrusion?

*We appreciate the reviewer for asking a complicated question here. The relationship between CTCF and PML-RARα has multiple levels. First, CTCF and PML-RARα have different binding preference, but some of the binding sites are closely clustered together. Therefore, observation in distance CTCF and PML-RARα could be appeared as co-localized, but in close-in they are not. Second, many of the CTCF loops and PML-RARα loops do overlap extensively, however, there is no clear comprehension. Lastly, it is at the domain level, where we have shown the majority of the PML-RARα binding and looping were in between CTCF domains for “stitching” the separate CTCF domains together, as we shown in Figure 2e-f and Figure S2a-b.*

*Can PML-RARα blocks loop extrusion? This is also a very interesting question, and it is highly possible. Unfortunately, the data in our current study would not be able to address this question. Certainly, this is an attractive question for us and others in future studies.*

3. Similar to the above comment, I really hope the authors can discuss whether non-CTCF-mediated loops (such as PML/RARa, YY1, or ZNF 143) are more transient than CTCF loops. I feel this discussion will be important for this field.

*We agree with the reviewer completely. CTCF is well known for its function in mediating chromatin folding architecture, and it can be used as a reference to relate other chromatin architecture proteins. In this study, although we directly compared CTCF and PML-RARα in PR9+Zn cells, we do not have specific data to assess the dynamics to conclude if PML-RARα is more transient than CTCF or not. However, based on the abundance of the PML-RARα binding intensity and loop frequency (Figure 1 and 2), we speculate that PML-RARα-mediated loops would be as strong as CTCF if not stronger. Accordingly, we have proposed that PML-RARα looping could alter the original CTCF-defined chromatin architecture into more of condensed conformation (Figure 2h, Figure S2g).*

4. Minor: regarding differential gene expression, the authors need to give significance (such as DESeq). It seems that right now they only rely on RPKM counts.

*We thank the reviewer for a very good poion here. For differential gene expression analysis, we used edgeR, a bioconductor software package for examining differential expression between count data (Robinson et al., Bioinformatics. 2010) that calculate log2 ratio and p value. In the revised manuscript, we included the differential expression data in supplement Table S4.*

One last comment, I really appreciate that the authors have released their data on GEO pre-publication. This is rare and I applauded the authors for the effort to keep their research transparent.

**Second round of review**

**Reviewer 2**

The authors have sufficiently addressed my questions.
